# Underpinnings of entangled ethnical and gender inequalities in obesity in Cochabamba-Bolivia: an intersectional approach

**DOI:** 10.1186/s12939-019-1062-7

**Published:** 2019-10-15

**Authors:** Yercin Mamani Ortiz, Per E. Gustafsson, Miguel San Sebastián Chasco, Ada Ximena Armaza Céspedes, Jenny Marcela Luizaga López, Daniel Elving Illanes Velarde, Paola A. Mosquera Méndez

**Affiliations:** 1 Biomedical and Social Research Institute, Faculty of Medicine, San Simon University, Cochabamba, Bolivia; 2 Department of Epidemiology and Global Health, Umeå University, Umeå, Sweden

**Keywords:** Obesity, Gender, Ethnic, Intersectionality, Health inequality, Decomposition analysis, Bolivia

## Abstract

**Background:**

Social inequalities in obesity have been observed not only by gender but also between ethnic groups. Evidence on combined dimensions of inequality in health, and specifically including indigenous populations, is however scarce, and presents a particularly daunting challenge for successful prevention and control of obesity in Bolivia, as well as worldwide.

**Objective:**

The aims of this study were i) to examine intersectional inequalities in obesity and ii) to identify the factors underlying the observed intersectional inequalities.

**Methods:**

An intersectional approach study was employed, using the information collected in a cross-sectional community-based survey. The sample consisted of youth and adults with permanent residence in Cochabamba department (*N* = 5758), selected through a multistage sampling technique. An adapted version of the WHO-STEPS survey was used to collect information about Abdominal obesity and risk factors associated. Four intersectional positions were constructed from gender (woman vs. men) and ethnic group (indigenous vs. mestizo). Joint and excess intersectional disparities in obesity were estimated as absolute prevalence differences between binary groups, using binomial regression models. The Oaxaca-Blinder decomposition was applied to estimate the contributions of explanatory factors underlying the observed intersectional disparities, using Oaxaca command in Stata software v15.1.

**Results:**

The prevalence of abdominal obesity had a higher prevalence in mestizos (men 35.01% and women 30.71%) as compared to indigenous (men 25.38% and women 27.75%). The joint disparity was estimated at 7.26 percentage points higher prevalence in the doubly advantaged mestizo men than in the doubly disadvantaged indigenous women. The gender referent disparity showed that mestizo-women had a higher prevalence than indigenous-women. The ethnic referent disparity showed that mestizo-men had a higher prevalence than indigenous men. The behavioural risk factors were the most important to explain the observed inequalities, while differences in socioeconomic and demographic factors played a less important role.

**Conclusion:**

Our study illustrates that abdominal obesity is not distributed according to expected patterns of structural disadvantage in the intersectional space of ethnicity and gender in Bolivia. In the Cochabamba case, a high social advantage was related to higher rates of abdominal obesity, as well as the behavioural risk factors associated with them.

## Background

The prevalence of obesity has risen to epidemic proportions worldwide [1–3]. This also applies to many Latin American countries, which over the last decade have experienced a remarkable increase in obesity, presumably associated to changes in lifestyle and demographics as nations transition from low-income to middle-income countries [1–3]. According to the World Health Organization (WHO), about 60% of Latin American population is overweight or obese [4]. While similar patterns have been reported in the Andean region in South America (men = 56.8% and women = 61.2%) [4], a slighter higher prevalence have been reported in Bolivia (60–63%) [5–7]. Consequently, the control and prevention of obesity is a priority in the Bolivian National Non-communicable Disease policy [8, 9].

The widespread social inequalities present a particularly daunting challenge for successful prevention and control of obesity In Bolivia as well as worldwide. For example, the increase in obesity has affected women more than men in Bolivia and in other Latin American countries [1, 4, 7, 8, 10]. These gender inequalities have been attributed to biological [11, 12], behavioral and social conditions, such as higher level of physical activity among men [7, 13], unemployment among women [14, 15], and the influence of gender roles in the choice of occupation [16, 17]. Social inequalities in obesity have been observed also by ethnic group [7]. In this case, obesity seems to be affecting more mestizo (people of mix European and Amerindian descendants) than indigenous [18–21].

.As is evident by the studies cited above, research on various kinds of social inequalities in obesity, in Bolivia as well as internationally, has so far been dominated by approaches where each axis of inequality – such as gender or ethnicity – are understood, analyzed and interpreted as independent and disentangled from each other [22]. Such simplified approaches to the complex phenomenon of social inequalities in health have however been increasingly challenged within global and public health, spurred by notions from intersectionality theory [23, 24]. Intersectionality theory took shape within social sciences in the late 1980s and involves an explicit recognition of the compound nature of multiple intersecting inequalities [22, 25, 26], but has only in recent years started to emerge as a novel approach to health inequalities within public health research and policy [22, 27–29].

In the case of Bolivian health and social policy, an emerging pressing concern is that particularly disadvantaged indigenous women is the group who fares the worst when it comes to health care access and social conditions such as quality of education, living wage and availability of food [9, 20]. This observation illustrates the entanglement of gender and ethnicity in the Bolivian context, and that approaches considering gender and ethnic inequalities as separate and disentangled may not be optimal as guidance for policy [8]. Therefore, understanding how ethnicity and gender intersect or combine to affect obesity would be particularly helpful to support the development of appropriate public health policies [30].

Intersectionality has to date mostly been applied to qualitative research but have become increasingly popular among quantitative public health researchers who are interested on analyzing inequalities in multiply marginalized populations [27, 29, 30]. To guide this line of research, Bauer [22] has formulated two complementary goals to intersectional, or compound, inequalities in health; first, to examine the health consequences of social positions of multiple advantage and disadvantage i.e. the pattern of intersectional inequalities; and second, to detail the processes whereby the social positions are expressed in population patterns of health. As an implementation of the first goal, Jackson et al. [31] have proposed an approach that considers the concept of a joint disparity, based on comparing absolute differences between dually as well as singly advantaged and disadvantaged groups. For the second goal, Blinder-Oaxaca decomposition [32, 33] has recently been suggested and exemplified as an appropriate method to disentangle the role of indicators representing social processes underpinning the inequalities [33, 34].

The present paper seeks to shed light on inequalities in obesity, in the intersectional space of gender and ethnicity, in Bolivia. The specific goals are i) to estimate the intersectional inequalities in obesity and ii) to identify the factors underlying the observed intersectional inequalities.

## Methods

### Design and study population

This study used data collected in a cross-sectional survey using WHO-STEPs approach [35] conducted in Cochabamba department, Bolivia, between 2015 and 2016 [7]. The target population included all residents in Cochabamba aged 19 and older [7]. A systematic random sampling procedure representative of municipalities, primary health care service areas, and communities was employed with 85.45% responding to the survey, resulting in a sample of *N* = 10,704 (refer to the other article or other document for details about the STEP approach and the content areas of the questionnaire) [7]. For the present study, all participants with valid responses on gender and ethnicity were eligible for inclusion. Those who did not identify themselves as Andean indigenous or mestizo (*N* = 109 African-American, whites or foreigners) and cases in which the information on key variables (e.g. income or other explanatory factors to the inequalities) were missing (*N* = 4837), were excluded from the analysis. After exclusions, the effective sample for the present analysis comprised *N* = 5758 individuals.

### Measures

#### Outcome: abdominal obesity

Abdominal obesity measured by waist circumference was chosen as health outcome as it has been found to be a more accurate predictor of cardio-metabolic risk than the general obesity measured by Body Mass Index (BMI) [36, 37]. Waist circumference was measured at the narrowest point between the lower costal border and the iliac crest using a constant-tension tape. Measurement of each individual was performed by trained health personnel as part of the application of the WHO-STEP survey. Following the WHO guidelines [8] abdominal obesity was defined as a waist circumference of > 90 cm in men and > 80 cm in women [38, 39].

#### Exposure: intersectional positions by ethnicity and gender

Gender and ethnicity identity were based on the information collected by the survey. Gender included the categories women [1] and men (0). Self-reported ethnicity was derived from the question: What ethnic group do you belong to? With the possibilities of Quechua, Aymara, mestizo, African-American, white or foreigner. For the purpose of this paper we included indigenous (Quechua and Aymara) to refer to pre-Columbian people in the Andean region of South America and their descendants, also known as Amerindians [1]; and mestizos (0) to refer to people of mix European and Amerindian descendants [40].

Based on the analytical approach suggested by Jackson et al. [31], gender and ethnicity were combined to form four mutually exclusive intersectional social positions. These intersectional social position may be reflected in disparate social processes of privilege or advantage, and conversely in oppression or disadvantage, which in turn can become expressed in varying degrees of health benefits [22]. The groups include: i) the dually disadvantaged group of indigenous women; ii) the dually advantaged group of mestizo men, and the singly disadvantaged groups of iii) indigenous men and iv) mestizo women.

#### Explanatory factors

Explanatory factors for the intersectional health inequalities included variables with known or possible links to obesity and to gender/ethnic [10, 19, 33, 37, 41] inequalities, and were grouped into:

1. Sociodemographic factors:
  - Age categorized into four groups according to the Global Burden of Disease-GBD: 18–29 (0), 30–44 (1), 45–59 (2), and ≥ 60 years (3) [42].
  - Place of residence classified according to the 5 socio-demographic regions of Cochabamba: Andean (0), Southern cone (1), Central Valley (2), Tropic (3) and High Valley (4).
  - Marital status categorized as: never married (0), currently married (1) and cohabitate or widowed/separated (2).
2. Socioeconomic factors:
  - Level of education categorized into four groups: no formal schooling (1), primary school (2), secondary school (3) and higher education (0).
  - Occupation classified into six groups: Students (0), self-employed (1), employed (2), housewife or homemaker (3), retired (4), and unemployed (5).
  - Monthly household income (an estimate based in the national minimum wage-NMW) categorized as: Less than a NMW (1), between 2 to 4 NMW (2), 5 or more NMW (0).
  - Health Insurance coverage classified as (1) if the individual has insurance and (0) otherwise.
3. Behavioral risk factors:
  - Smoking was categorized as current smoker [1] or non-smoker (0) in accordance WHO-STEPs survey manual [42].
  - Alcohol consumption was explored through the items adopted from “Alcohol Use Disorders Identification Test (AUDIT)” [43] included in the STEPS survey, which collect information about three different aspects: amount, frequency, and patterns of drinking. Participants were classified as harmful use of alcohol (1 = present; 0 = absent) if heavy drinking episodes (6 or more standard drinks when alcohol is used) were reported either: a) once a month or more in the last 12 months; b) twice or more in the past 30 days; or c) if drank until getting drunk at least once in the past 7 days [42].
  - Low consumption of fruits and vegetables was classified according to the WHO STEPS. I.e. eating less than five servings or approximately 200 g of fruits and vegetables per day for both food groups were classified as at risk (1); otherwise as adequate (0) [42].
  - Physical activity was measured through the items from “the Global Physical Activity Questionnaire” included in the STEPS survey, which collect information about four different aspects: physical activity at workplace, during recreation time, while travelling, and during resting time. This variable was categorized according to the Metabolic Equivalent of Task (MET) as: low (insufficient) physical activity (1) for values lower than 600 MET-minutes per week; and appropriate physical activity (0) for values higher than 600 MET-minutes per week [42].

### Statistical analysis

#### Drop-out analyses

Due to complete case analysis, drop-out analyses were performed to investigate potential selection bias introduced by the internal drop-out. Missingness was defined as (1) (individuals who had missing values in any of the used variables and therefore were excluded, *N* = 4837); and complete cases as (0) (*N* = 5758). The missingness variable was regressed on each variable used in the study in separate simple logistic regression models. The drop-out was significantly (*P* < 0.01) predicted by older (OR = 1.55), women (OR = 1.46), less educated (OR = 1.14), homemakers (OR = 1.32) and living in the central valley (OR = 1.12).

### Measurement of intersectional inequalities

In accordance with the procedure illustrated by Jackson et al. [31] the joint, referent and excess intersectional disparities were estimated as absolute prevalence differences between binary groups, using binomial regression models by the following comparisons:

1. Mestizo men (MM = 0) vs indigenous women (IW = 1) referred as **joint disparity** compares outcomes among the dually advantaged/disadvantaged categories, respectively; thus capturing the health inequality between the most advantaged and the most disadvantaged social positions.
2. Mestizo men (MM = 0) vs mestiza women (MW = 1) referred as **referent gender disparity**. This comparison evaluates obesity disparities among mestizos (those who does not face ethnic discrimination) and illustrates how the outcome is patterned by gender disadvantage.
3. Mestizo men (MM = 0) vs indigenous men (IM = 1) referred as **referent ethnic disparity.** This comparison evaluates the obesity disparities among men (those with an advantage in relation to gender) and describes how the outcome is patterned by racial disadvantage.
4. The **excess intersectional disparity** was calculated as the difference between the joint disparity and the sum of ethnicity disparity plus gender disparity**.** This corresponds to a measure of additive interaction and quantifies by how much the joint disparity exceeds the sum of two referent disparities, thus capturing to which degree the disparity for the dually disadvantaged groups surpasses what would be expected if the two axes of inequality were considered as independent axes of inequality.

The disparities are additive measures and thus reflect absolute inequalities indicating the absolute gains in the population outcome that would be achieved if the disparity were removed. For the interpretation of the intersectional disparities, the referent and excess disparities reported both in absolute terms as prevalence differences, as well as percentage of the joint disparity.

### Contribution of social determinants to intersectional inequalities

The Oaxaca-Blinder Decomposition was applied to estimate the contributions of explanatory factors underlying the observed joint and referent intersectional disparities. The principle of the decomposition method is based on two regression models that are run independently for each of the groups to be compared (Adv = Advantaged and Dis = Disadvantaged), and that for the present study could be expressed as follows:

$$ {\mathrm{Obesity}}^{\mathrm{Adv}}=\beta {\mathrm{X}}^{\mathrm{Adv}}+\left(\upvarepsilon \mathrm{Adv}\right) $$

and

$$ {\mathrm{Obesity}}^{\mathrm{Dis}}=\beta {\mathrm{X}}^{\mathrm{Dis}}+\varepsilon \mathrm{Dis} $$

Where, β is the coefficient that includes the intercept; X are the explanatory variables, and ε is the error. The obesity gap between the two groups is then decomposed into the part that is due to difference in the means of the explanatory factors (explained component) and to differences in the effects (unexplained component). The obesity gap could be expressed as follow:

$$ {\Delta^{\mathrm{Dis}\hbox{-} \mathrm{Adv}}}_{=}\left({\overline{\mathrm{X}}}_{\mathrm{Adv}}-{\overline{\mathrm{X}}}_{\mathrm{Dis}}\right)\ {\beta}_{\mathrm{Dis}}+{\overline{\mathrm{X}}}_{\mathrm{Adv}}\left({\beta}_{\mathrm{Adv}}-{\beta}_{\mathrm{Dis}}\right) $$

Where the first term of the equations [($\overline{\mathrm{X}}$_Adv_ – $\overline{\mathrm{X}}$_Dis_) β_Dis_], corresponds to the observable difference in the means of explanatory variables weighted by the coefficient of the advantaged/disadvantaged group (explained component). The second term [($\overline{\mathrm{X}}$_Adv_ (β_Adv_ – β_Dis_)] represent the differences in the coefficients of the explanatory variables, weighted by mean of the disadvantaged/advantaged group (unexplained component) [32, 44]. Corresponding extensions of Oaxaca decomposition analysis have also been developed to the nonlinear case when using a binary outcome [45], such as in this study.

The analysis were conducted using Oaxaca command in STATA software v15.1 [34]. In the present study, we decomposed the obesity gaps for the following three comparisons: 1) mestizo men vs mestizo women; 2) mestizo men vs indigenous men, and 3) mestizo men vs indigenous women. All the explanatory factors described above were included in the model. Collinearity was evaluated by variance inflation factor analysis (Mean VIF: 1.23) [46].

To facilitate the interpretation of the contributions in the decomposition analyses, the group with higher prevalence of obesity (socially advantaged group of mestizos) was considered as the disadvantaged group. First, the proportion of obesity and the group differences in proportion for each of the performed comparisons were calculated. Second, we estimated the absolute independent contribution of each of the explanatory factors (on the same scale as the health gap; i.e. proportion difference). The relative contributions (%) were calculated with respect to the absolute total health gap for the explained and unexplained components, and relative to the absolute explained component for the contributions of each explanatory factor**.**

## Results

### Descriptive statistics across intersections

Table 1 describes the distribution of explanatory factors in the study population in each of the four intersectional social position by gender and ethnic group. Overall, indigenous were less obese and showed healthier habits than mestizo but reported less favorable socioeconomic conditions. The average waist circumference was higher in mestizos (90.25 ± 0.49 in men and 88.28 ± 0.46 in women) than in indigenous (87.69 ± 0.32 in men and 87.96 ± 0.28 in women). Likewise, mestizo had a higher prevalence of abdominal obesity (men 35.01% and women 30.71%) as compared to indigenous (men 25.38% and women 27.75%). (Table 1).

**Table 1:** Descriptive Statistics of all variables in the total sample and by intersectional positions of gender and ethnicity^a^

Variable	Women		Men		Both	
	(*N* = 3182–55.26%)		(*N* = 2576–44.74%)		(N = 5758)	
	Indigenous	Mestizo	Indigenous	Mestizo	Indigenous	Mestizo
n	2166	1016	1622	954	3182	2576
%	57.18	51.57	42.82	48.43	55.26	44.74
Waist circumference						
Mean and SE (cm)	87.96 ± 0.28	88.28 ± 0.46	87.69 ± 0.32	90.25 ± 0.49	87.85 ± 0.21	89.24 ± 0.33
% abdominal obesity	27.75	30.71	25.83	35.01	26.93	32.73
Sociodemographic factors						
Age group (years)						
18–29	41	52.46	32.43	44.34	37.33	48.53
30–44	28.44	26.48	27.31	26.94	27.96	26.7
45–59	14.82	14.07	18.87	17.08	16.55	15.53
≥ 60	15.74	6.99	21.39	11.64	18.16	9.24
Place of Residence						
Andean	11.96	4.33	10.78	5.35	11.46	4.82
Southern cone	8.08	1.87	8.88	2.3	8.42	2.08
Central Valley	34.16	47.64	33.42	52.2	33.84	49.85
Tropic	17.13	15.75	18.25	18.03	17.61	16.85
High Valley	28.67	30.41	28.67	22.12	28.67	26.4
Marital Status						
Never married	23.13	28.94	23.67	36.48	23.36	32.58
Currently married Cohabitate	67.13	63.58	68.99	58.28	67.93	61.02
Widowed or Separated	9.74	7.48	7.34	5.24	8.71	6.4
Socioeconomic factors						
Education Level						
No formal schooling	12.14	2.17	7.09	1.15	9.98	1.68
Primary school	49.49	29.63	47.53	26.21	48.65	27.97
Secondary school	28.63	47.83	35.33	49.06	31.49	48.43
High education	9.74	20.37	10.05	23.58	9.87	21.93
Job status/occupation						
Student	9.27	14.86	8.51	13.52	8.95	14.21
Self-employed	31.86	33.27	70.46	55.97	48.39	44.26
Employed	9.6	15.06	15.17	24.32	11.99	19.54
Homemaker	47.05	34.45	0.62	0.63	27.16	18.07
Retired	1.11	1.28	3.82	3.88	2.27	2.55
Unemployed	1.11	1.08	1.42	1.68	1.24	1.37
Monthly household income						
Less than a NMW	39.06	24.21	34.03	18.13	36.91	21.27
Between 2 to 4 NMW	50.14	59.45	52.9	58.91	51.32	59.19
More than 5 NMW	10.8	16.34	13.07	22.96	11.77	19.54
Health Insurance						
Yes	14.82	15.75	17.08	17.19	15.79	16.45
No	85.18	84.25	82.92	82.81	84.21	83.55
Behavioral risk factors						
Current smokers	2.95	5.12	25.28	23.79	12.51	14.16
Alcohol consumption	36.52	39.67	57.71	61.64	57.71	61.64
Low Fruit and Vegetables intake	78.35	71.36	80.83	72.43	79.41	71.88
Physical activity						
Low	66.39	75.3	44.45	55.55	57	65.74
Moderate	29.69	22.44	39.46	37.32	33.87	29.64
High	3.92	2.26	16.09	7.13	9.13	4.62

^a^Numbers are column percentages within each variable, unless otherwise noted.

The distribution by sociodemographic factors was similar in both groups, with some exceptions: the indigenous group had a higher proportion of > 60 years (18.16%) and people living in the Andean (11.46%) and Southern Cone (8.42%) than mestizos. (Table 1).

In relation to distribution by socioeconomic factors, a higher proportion of mestizos (21.93%) reached the highest level of education, while the indigenous have a higher proportion of people with no formal education (9.98%). A larger proportion of mestizo belong to the highest level of household income (19.54%), while indigenous have a higher proportion of people in the lowest level (9.98%). Regarding employment, a very high proportion of women in both ethnic groups were homemakers, with a higher prevalence of this occupation among indigenous (47.05% indigenous and 34.45% mestizo). A very low proportion of individuals in both groups had health insurance coverage (< 18%). (Table 1).

Most of the behavioral risk factors were more prevalent in the mestizo group, i.e. smoking (14.16% vs 12.51%), current alcohol consumption (61.64% vs 57.71%) and low physical activity (65.74% vs 57%). However, the low fruits and vegetable consumption was more prevalent among the indigenous (79.41% vs 71,88%). (Table 1).

### Gender and ethnic intersectional inequalities in obesity

Table 2 reports the joint, referent and excess disparities between mestizo and indigenous including the percentage of the joint disparity attributable to each component. The *joint disparity* showed the obesity prevalence was 7.26 percentage points higher in the doubly advantaged mestizo men than in the doubly disadvantaged Indigenous women. The *gender referent disparity* showed that mestizo women had 4.30 percentage points higher prevalence than indigenous-women, accounting for 59% of the joint disparity. *The ethnic referent disparit*y showed that mestizo men had 9.18 percentage points higher prevalence than indigenous men, representing 126% of the joint disparity. The resulting *excess intersectional disparity* was 6.22% representing − 86% of the joint disparity. Thus, the 7.26% joint disparity in obesity was to a main degree due to ethnic differences alone, rather than gender-related inequalities. Moreover, the doubly disadvantaged group of indigenous women, while having a more than 7 percentage points lower prevalence of obesity than mestizo men; nevertheless, had a markedly higher obesity prevalence - 6 percentage points - than would be expected from the fact that they were women on the one hand, and of ethnic minority on the other.

**Table 2:** Joint disparity and component disparities for the abdominal obesity prevalence

	Joint disparity (MM-IW)	Gender reference disparity (MM-MW)	Ethnicity reference disparity (MM-IM)	Excess intersectional disparity (MM-IW)-(MM-IM)-(MM-MW)
Abdominal obesity (%)				
Absolute difference (95%IC)	7.26 (3.64–10.88)	4.30 (0.09–8.52)	9.18 (5.42–12.94)	- 6.22 (1.1–11.34)
% Attributable	–	59.00	126.00	−86.00

### Decomposition of abdominal obesity gaps between intersectional groups

Table 3 presents the absolute and relative contributions of each explanatory factor as well as the joint contribution of groups of explanatory factors to the abdominal obesity gaps. The included factors jointly explained 51.08% of the joint disparity, 83.65% of the gender referent disparity, but only 11.72% of the ethnic referent disparity.

**Table 3:** Oaxaca-Blinder Decomposition of the gap in the prevalence of abdominal obesity comparing advantaged and disadvantaged groups

	Joint disparity			Gender referent disparity			ethnic referent disparity		
	Mestizo Men (group 1)			Mestizo Men (group 1)			Mestizo Men (group 1)		
	Indigenous Women (group 2)			Mestizo Women (group 2)			Indigenous Men (group 2)		
Model estimates	Coefficient	p	%	Coefficient	p	%	Coefficient	p	%
AO (group 1)	0.35	< 0.01	35.01	0.35	< 0.01	35.01	0.35	< 0.01	35.01
AO mean (group 2)	0.277	< 0.01	27.75	0.307	< 0.01	30.71	0.258	< 0.01	25.83
Difference	0.073	< 0.01	7.26	0.043	0.04	4.3	0.092	< 0.01	9.18
Explained fraction	0.037	0.01	51.08	0.036	0.02	83.65	0.011	0.32	11.72
Unexplained fraction	0.036	0.08	48.92	0.007	0.76	16.35	0.081	< 0.01	88.28
Factor contributions									
Sociodemographic factors									
Age group									
30–44	− 0.004	0.39	−9.49	0.001	0.82	2.46	−0.001	0.84	−13.62
45–59	0.008	0.11	20.7	0.01	0.06	27.00	−0.009	0.43	−81.01
≥ 60	−0.011	< 0.01	−29.7	0.012	< 0.01	34.48	−0.047	0.21	− 438.85
			−18.49			63.94			− 533.49
Residence									
Southern cone	−0.005	0.07	−14.5	0	0.63	−0.71	−0.009	0.29	−80.70
Central Valley	0.024	< 0.01	65.42	−0.002	0.53	−4.86	0.009	0.47	83.05
Tropic	0.002	0.55	4.86	0.001	0.55	2.53	−0.001	0.89	−4.68
High Valley	−0.002	0.39	−6.4	0.005	0.34	13.20	−0.003	0.55	−26.30
			49.38			10.16			−28.64
Marital Status									
Currently married Cohabitate	−0.004	0.10	−11.93	−0.002	0.22	−6.59	− 0.005	0.45	−46.43
Widowed or Separated	−0.002	0.23	−6.44	−0.002	0.23	−4.99	−0.003	0.34	−31.63
			−18.37			− 11.58			−78.05
Subtotal contribution of sociodemographic factors			12.52			62.52			− 640.18
Socioeconomic factors									
Education Level									
No formal schooling	0.006	0.25	16.83	0.002	0.16	6.79	0.028	0.15	264.22
Primary school	−0.005	0.57	−12.42	0	0.82	0.76	0.027	0.19	248.77
Secondary school	−0.002	0.77	−5.23	0	0.62	−1.31	−0.011	0.29	−98.81
			−0.81			6.25			414.18
Job status									
Self-employed	0.044	< 0.01	119.54	0.036	< 0.01	100.07	−0.036	0.27	− 337.47
Employed	0.019	0.04	50.33	0.009	0.09	25.08	0.017	0.32	153.76
Homemaker	−0.073	0.01	− 197.88	− 0.066	< 0.01	− 183.66	0	0.96	0.39
Retired	0.005	0.04	14.74	0.004	0.14	10.07	0	0.94	1.01
Unemployed	0.001	0.28	3.74	0.001	0.31	3.99	0.001	0.61	8.02
			−9.52			−44.46			− 174.28
Monthly household income									
Less than a NMW	0.014	0.05	37.04	0.002	0.42	5.2	0.021	0.21	195.37
Between 2 to 4 NMW	−0.001	0.63	−3.35	0	0.83	−0.23	−0.003	0.42	−24.2
			33.69			4.97			171.17
Health Insurance	0	0.86	−0.32	0	0.62	0.76	0	0.94	0.17
Subtotal contribution of sociodemographic factors			23.04			−32.48			411.24
Behavioral risk factors									
Smoking	0.012	0.07	33.69	0.007	0.26	19.60	−0.002	0.51	−14.39
Alcohol consumption	0.017	< 0.01	45.95	0.025	< 0.01	70.30	0.004	0.24	36.64
Low Fruit and Vegetables intake	0.004	0.03	9.58	0	0.95	0.05	0.006	0.23	58.54
Physical activity									
Low	−0.003	0.07	−8.44	−0.003	0.33	−7.44	0.024	0.17	220.85
Moderate	−0.006	0.001	−16.34	−0.005	0.24	−12.54	0.003	0.39	27.31
			−24.78			−19.98			248.16
Subtotal contribution of sociodemographic factors			64.44			69.97			328.95

The higher prevalence of obesity in mestizo men compared with indigenous women (*joint disparity*) was mainly explained by behavioral risk factors, which alone stood for 64.44% of the explained portion of the health gap. Alcohol consumption and current smoking were the dominant contributing factors as they were also more frequent in mestizo men than in indigenous women (Table 1). Socioeconomic factors were the second most important group of variables explaining the obesity gap (23.04%), with income being the most important individual contributor. Sociodemographic factors jointly contributed to the obesity gap to a moderate degree (12.52%), with place of residence being the most important contributor (Table 3).

In contrast, when it comes to explaining the higher prevalence of abdominal obesity in mestizo men compared to mestizo women (gender referent disparity), behavioral risk factors (69.97%) and sociodemographic variables (62.52%) were of similar importance. Alcohol consumption (70.30%) and age (63.94%) were the most important contributors to explain the obesity gap, followed by smoking (19.6%) and place of residence (10.16%) (Table 3). This is confirmed by the description in Table 1, where mestizos men had a higher proportion of alcohol consumption (61.64%); but a lower proportion of people > 60 years (11.64%) and current smokers (23.79%) compared with indigenous men. Conversely, the joint contribution of socioeconomic factors was offsetting the observed inequality (− 32.48% contribution), as none of the factors contributed much to the explanation of the gap but instead, as the job status that displayed a large offsetting contribution (− 44.46%). (Table 3).

The estimates for the decomposition of the *ethnic referent disparity* between mestizo and indigenous men where unstable, as reflected in the small and non-significant fraction explained by the set of factors, but the results are nevertheless reported here for the sake of completeness. Socioeconomic factors (411.24%) and behavioral risk factors (328.95%) were the most important. Here, the level of education and income, as well as the prevalence of physical activity, were the most important contributors to explain the obesity gap (Table 3). This is mirrored by the description in Table 1, where high levels of education and income were more frequent in mestizo men while the prevalence of physical activity was higher in indigenous men. The small explained portion of the obesity gap was due to the offsetting contribution of sociodemographic factors, specifically age which made a large negative contribution. As it can be seen in Table 1, this offsetting contribution of age is due to the higher frequency of older people among indigenous men (> 60 years 21.39%). (Tables 1 and 3).

## Discussion

Despite the calls for intersectional global health approaches in low- and middle-income countries [23, 24], to the best of our knowledge, this is the first study estimating and explaining obesity inequalities in the intersection between gender and ethnicity in Bolivia and Latin America. First, we found that dually and singly disadvantaged groups (indigenous women, indigenous men and mestizo women) were less obese than the dually advantaged group (mestizo men), meaning that in this study population, the socially advantaged were in fact disadvantaged with respect to health. Second, the lower prevalence of obesity in the doubly disadvantaged group of indigenous women was mainly due to ethnic differences alone. However, they had higher obesity than we might expect from considering both genders alone and ethnicity alone. Third, the results highlighted intersectional disparities in health behaviors as a ubiquitously important factor to explain the observed inequalities, while differences in socioeconomic and demographic factors played a less important role.

The finding of socially advantaged population being more obese have commonly been reported in low and middle-income countries [47] where obesity has been associated with higher socioeconomic levels and high level occupations [32, 48]. In our study, indigenous women, who have been historically considered as socially disadvantaged due to low levels of education and income as well as low level occupations [14], were less affected by obesity than the mestizo men. Our findings overall confirm the already reported inequalities in obesity to the disadvantage of mestizo in Bolivia [7] and expands previous research in the Latin-American region focusing on single axis of inequalities such ethnicity [7, 19, 21, 49] and gender [7, 50].

The lower prevalence of obesity found in the doubly disadvantaged group was mainly attributed to ethnic inequalities. Previous studies have pointed out that the indigenous population has a lower prevalence of obesity among other cardiovascular risk factors than mestizo population [7]. This difference could be related to the indirect effects of social disadvantage on their health behaviors. For example, indigenous population engage in high levels of physical activity, in women for example due to raising animals for self-consumption (sheep, goat, etc.) as part of their domestic activities, which in some cases involve walking long distances in steep terrain [17, 19–21]. Indigenous women also have a lower prevalence of other behavioral risk factors such as smoking and alcohol consumption commonly associated with the development of obesity [51, 52], which is considered inappropriate for women as part of moral regulations introduced by evangelical movements since the 1990s, especially strong among the indigenous communities [7, 53].

Moreover, despite that the dually disadvantaged group of indigenous women displayed lower obesity than the dually advantaged mestizo men; our findings showed that they still had higher obesity prevalence than we might expect from the fact that they were women on the one hand, and of ethnic disadvantage on the other, both facets of social disadvantage by themselves being protective against obesity. This pattern could reflect certain obesogenic exposures particular for this intersection of gender and ethnicity; for example, the high birth rates [54] specifically among indigenous women in Bolivia [18], where nearly half of the women are housewives as it is socially expected that they stay home taking care of child [55, 56], or possibly other forms of material and psychosocial disadvantage that may contribute to obesity through e.g. stress [57]. Moreover, despite the domestic work of indigenous women involving high levels of physical activity, it is still be less arduous than for the indigenous men who are not as bound to the confines of the home [56, 58].

Taken together, the seemingly paradoxical finding of overall low but nevertheless higher-than-expected obesity prevalence among indigenous women could be construed as reflecting a hidden form of inequality, resulting from dual social processes with counteracting effects on this aspect of health. In this case, the harmful consequences of pervasive social disadvantage on obesity appears to be partially hidden by the simultaneous presence of both a physically demanding life and the presence of gender norms that discourage indigenous women from engaging in other harmful health behaviors. These are undoubtedly additional facets of social disadvantage, oppression and marginalization, but which may be protective against obesity. These findings illustrate the contextually dependent, interacting and multifaceted structural roots of social processes that may shape complex population patterns in health, how social disadvantage and oppression may be expressed in paradoxical ways when it comes to health, and the unique value of intersectional perspectives to uncover such phenomena [23] . On the other hand, this difference between mestizo and indigenous people might be related to the type of diet or food preparation; further research is therefore needed to explain these differences [59].

Further clues to the social processes at play were suggested in the explanatory decomposition analyses of the joint and referent inequalities. Behavioral risk factors were consistently important to explain the gender and ethnic disparities. Overall, behavioral risk factors were more prevalent among men, especially in mestizo men, except for the low physical activity, which was more prevalent in women, especially mestizo women. These differences are likely a reflection of the social roles assigned to men in the Bolivian society; as described by other authors, men are disproportionally exposed to tobacco and alcohol consumption from an early age [60–62] as well as to physically demanding work, which in some cases continues up to the old age. This is particularly the case for elderly indigenous men who performing vigorous physical activity throughout their life until very advanced ages [14], which furthermore could explain the low prevalence of abdominal obesity in indigenous men, especially in the elderly group.

Socioeconomic and demographic factors were of less universal importance to explain the inequalities, except for level of education and age which contributed to the explanation of the ethnic and gender disparity respectively. For example, on explaining the lower prevalence of obesity among indigenous men compared to mestizo men, the higher rate of indigenous men with no formal schooling or only primary education may have played a role. Specifically, because of their low education, indigenous men are mainly involved in non-skilled manual occupations (like agricultural work) which involve vigorous physical activity, while in contrast their mestizo peers have a greater chance to achieve higher educational levels [16, 63] and therefore are mainly involved in non-manual occupations which are related with sedentary life styles [64].

Overall, the population patterns of abdominal obesity identified in this study highlight the value of considering intersectionality for the generation of public health policies that adequately prioritize subgroups in needs, in accordance with the “leave no one behind” agenda [23]. So far, health policies in Bolivia have generally emphasized interventions targeting the socially vulnerable or disadvantaged populations [8, 65], but in the case of abdominal obesity, the group with a double social advantage requires a high attention. More specifically, interventions taking into account the gender and ethnic intersection could be aimed at increasing levels of physical activity among mestizo men [10, 49, 63, 66] and indigenous woman as well as to reduce tobacco and alcohol consumption among both mestizo and indigenous men [13].

## Strengths and limitations

Our study is based on a representative population sample of Cochabamba Bolivia, the information was collected through a standardized process validated by WHO / PAHO, in addition to the application of innovative statistical approaches for the analysis of health inequities.

The drop-out analysis showed that older people, women, less educated, homemakers and residents of the central valley were more likely to not have complete data and therefore to be excluded from the complete case analyses. These signs of selection bias, in combination to the unknown extent of selection when it comes to participation in the study as a whole, means that caution should be applied when interpreting and generalizing the findings.

Concerning the analysis, Oaxaca decomposition can be viewed as a useful method to identify factors underlying inequalities, however, it cannot provide causal inference, a matter that is only reinforced by the cross-sectional nature of our data.

The individuals who did not answer the questions about their economic income were not included in this study, reducing the sample size and potentially introducing a degree of selection bias, as indicated by our drop-out analysis. Whereas this might bias the estimated overall prevalence of obesity, it is less likely that the main analyses building on group differences and decompositions are severely biased.

It should also be considered that the information about risk factors is self-reported, and measurement bias in some of the exposures could be introduced. Because, participants were wary and suspicious of answering some questions, especially those about alcohol and tobacco use (some persons scared of being judged or felt ashamed to answer affirmatively) their prevalence could have been underestimated. This in turn might have led to an underestimation of the role of these factors on explaining the inequalities.

The explained fraction of ethnic referent disparity was very small for traditional risk factors included in the WHO-STEPS approach used in our study, which are also the factors traditionally associated with abdominal obesity in almost all previous studies reviewed. This suggests the need to explore other factors not covered by the WHO STEPS questionnaire (i.e. stress, poverty, diet, etc.) and that could help to improve the understanding of this phenomenon, as well as the development of more specific strategies for these population groups.

The information collected was disaggregated by municipalities and sociodemographic macro-regions, but the specific rural/urban location of the participant communities was not available and therefore not included in the analysis. It is probable that occupation, the level of physical activity and dietary habits vary significantly between rural and urban indigenous and mestizo population groups missing therefore the potential contribution of rurality to the obesity inequality.

## Conclusions

Our study illustrates that abdominal obesity is distributed in a seemingly paradoxical pattern in the intersectional space of ethnicity and gender, where a high social advantage was related to higher rates of abdominal obesity. Nevertheless, the doubly disadvantaged group of indigenous displayed a higher prevalence of obesity than were to be expected, which we interpret as an expression of counteracting health effects of compound social disadvantage.

The behavioral risk factors were those that most influenced the explanation of inequities in the socially doubly advantaged group compared to the other subgroups. This can give guidance to health prevention programs that seek to improve health as well as equity in health. Importantly, from a public health perspective, our findings indicate that intervention strategies cannot be approached in the same way in all population subgroups, a key notion of intersectional public health. This observation requires prioritization of certain intervention groups, in order to reduce the compound gender and ethnic inequalities in health based on the contextual population patterns of abdominal obesity.

More broadly, the present study illustrates the added value of applying an intersectional perspective and analysis to public health. More studies on intersectionality are necessary to understand the complex interaction between gender and ethnicity on obesity, considering those non-traditional risk factors that can show a more complete panorama of this phenomenon and be able to generate more precise prevention and control strategies.

## Data Availability

The datasets supporting the conclusions of this article are available upon request.

## Abbreviations

AUDIT: Alcohol Use Disorders Identification Test
BHM: Bolivian Health Ministry
BMI: Body Mass Index
CVDs: Cardiovascular diseases
GBD: Global Burden of Disease
GPAQ: Global Physical Activity Questionnaire format
IM: Indigenous men
IW: Indigenous women
MET: Metabolic Equivalent of Task
MM: Mestizo Men
MW: Mestizo Women
NCD: Non-communicable diseases
NMW: national minimum wage
PAHO: Pan American Health Organization
WHO: World Health Organization
